# An effective enzyme-linked immunosorbent assay-based method for quantification of gibberellic acid in pecan (*Carya illinoinensis*)

**DOI:** 10.3389/fpls.2026.1771880

**Published:** 2026-06-24

**Authors:** Mohadeseh Jahanifard, Ming Yang, Amandeep Kaur, Lu Zhang

**Affiliations:** 1 Department of Horticulture and Landscape Architecture, Oklahoma State University, Stillwater, OK, United States; 2 Department of Biology, Oklahoma State University, Stillwater, OK, United States

**Keywords:** bark, bud, ELISA, GA quantification, woody plant

## Abstract

Enzyme-linked immunosorbent assay (ELISA) is a reliable and low-cost method for quantifying gibberellic acid (GA) in plants but applying this method to certain plant tissues is challenging due to tissue hardness and/or contents rich in metabolites. Pecan (*Carya illinoinensis*) tissues in the bud and bark are examples of such tissues. This study aimed to establish an effective ELISA-based GA quantification method for these pecan tissues. We tested how different amounts of the tissues and the weight ratios of a tissue to the solvent methanol affected the detection of GA. We also tested whether heat-drying and freeze-drying and long-term storage of the fresh materials in -80 °C affected the detection of GA. Our results showed that using 0.0125± 0.0005 gram (g) of heat-dried or freeze-dried tissues and a relatively low tissue-to-methanol ratio consistently produced high GA concentrations in ELISA detection, and the levels of GA were stable in pecan samples stored long-term at -80 °C. Our results also showed that GA concentrations in buds were higher than in the bark. These findings indicate that our method is effective in detecting GA in small amounts of pecan tissues, and heat-drying of either fresh or freezer-stored samples can produce satisfying results of GA quantification. Moreover, the GA concentrations in the wood and bark were found to be positively correlated with the GA concentrations in the bud, which help estimate most part of the variability of the GA concentration in the bud based on the GA concentration in the bark, as it may be challenging to experimentally determine the GA concentration in a small bud at an early developmental stage. The final spike-recovery test with GA (From ELISA kit) as spike and sample to sample spike confirmed that our sample extracts exhibited acceptable recovery percentages in low and high spike with range of 72-102% of recovery indicating that matrix effects and phenolic interference did not significantly affect the results.

## 1 Introduction

Phytohormone levels differ considerably across plant tissues, developmental stages, and environmental conditions. The hormonal dynamics are integral parts of signaling mechanisms in plant development and plants’ responses to environmental changes. Accurate determination of hormonal concentrations in small amounts of tissue is crucial to understanding the quantitative relationships between the levels of hormones and the expression levels of hormone-responsive genes. Hormonal quantification methods using small amounts of tissue (Šimura et al., 2018; Cao et al., 2022) and with a low cost and experimental simplicity are especially welcome when the same limited tissue samples may be subject to multiple investigations, such as gene expression, carbohydrate profiling, and GA quantification studies.

GA is a key phytohormone that regulates plant development and stress responses (Davis, 2004). Quantifying GA in different tissues in the shoot of woody perennials, such as pecan (*Carya illinoinensis*), may provide critical evidence for how GA affects the development of male and female flowers (Jia et al., 2018; Kaur et al., 2021). Liquid chromatography-tandem mass spectrometry (LC-MS/MS) is often used for quantifying plant hormones, including GA (Wang et al., 2020; Hirayama and Mochida, 2022; Cao and He, 2023). A recent study developed a rapid and sensitive high-throughput ultra-performance liquid chromatography tandem mass spectrometry (UPLC-MS/MS) method capable of quantifying phytohormones, including GA, from 0.01 g of plant tissue (Cao et al., 2020). However, the LC-MS/MS approach in general requires expensive instrumentation and specialized technical skills, likely to prevent its use in a small laboratory setting.

The chemical complexity and large quantities of plant cell wall and intracellular components pose an inherent challenge to hormone extraction, often requiring adjustments to extraction solvents and cleanup procedures for a particular tissue. Studies across various species have shown that tissue-specific chemical composition influences extraction yield and reliability (Vrobel, 2023). Previously, different solvents have been used in hormone extraction from plant tissues, with non-polar solvents (e.g., ethyl acetate and dichloromethane) showing marked differences in extraction efficiency between fresh and dried materials, and polar solvents (e.g., a mixture of methanol and water) not showing such differences (Almeida Trapp et al., 2014).

Enzyme-linked immunosorbent assay (ELISA) is a low-cost method for quantification of GA in plant tissues without needing expensive equipment and specialized technical skills. The protocols for conducting ELISA are available from ELISA kit manufacturers, but these protocols lack critical information on the sample processing method for hard samples such as the phloem containing bark and the buds of pecan. In this report, we present a protocol for extracting GA from a small amount of tissues from pecan bark or buds, followed by quantification of GA using ELISA. Our results demonstrated that the method was effective for GA quantification with simply heat-dried samples. Our results also showed that GA concentrations in buds and bark on the same tree were positively correlated, providing a basis for correlative analysis of GA concentrations in different parts of the pecan.

## 2 Materials and methods

### 2.1 Materials

All plant materials were collected from two Pawnee pecan trees planted in 1994, located at the Cimmaron Valley Research Station in Perkins, Oklahoma, USA, a property of Oklahoma State University. These materials were collected three times, first in late July 2024 and then two times in late March 2025. To account for spatial variability, 20 current-year shoots were sampled per collection from four sides of the two canopies (north, south, east, and west). To minimize hormone degradation (Cao et al., 2017, 2022; Šimura et al., 2018; Katyayini et al., 2020), the samples were transported from the orchard to the laboratory on dry ice in a cooler and stored at −80 °C upon arrival, except for one treatment in the final experiment, which was heat-dried immediately after arrival.

### 2.2 Sample drying and grinding procedures

Before any heat or freeze-drying process all current shoot samples have been separated into wood, bark and bud. Later, sample drying was carried out in a Fisherbrand Isotemp General Purpose Heating and Drying Oven (Thermo Fisher Scientific, Waltham, MA, USA) at 60 °C for four days, or in a benchtop Harvest Right Stainless-Steel Pro Freeze Dryer (Harvest Right, Salt Lake City, UT, USA) at −42 °C and 0.1 mbar for two days.

**Figure 1:**
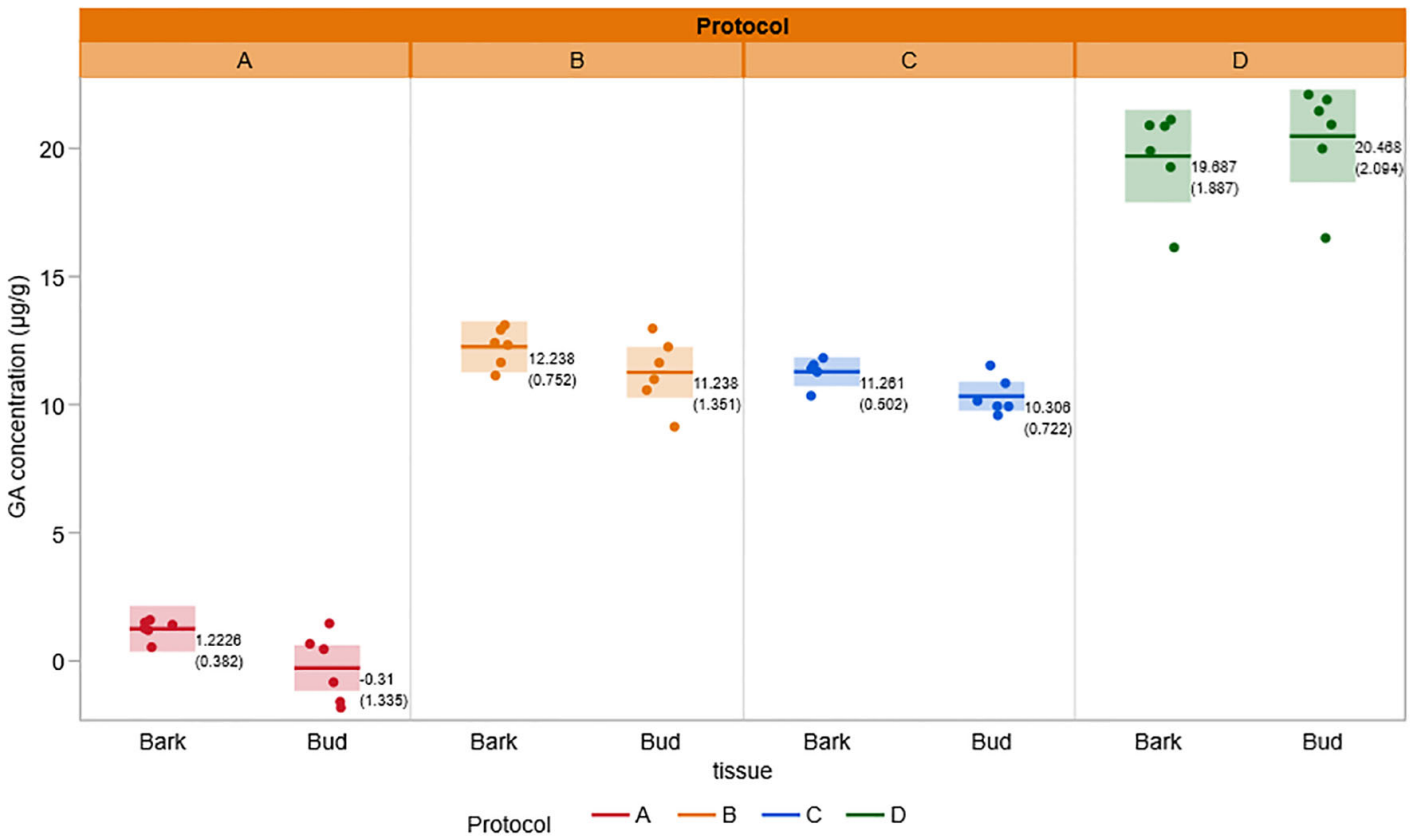
Effect of extraction protocols (sample weight) and solvent ratio on GA concentration (µg/g) in different dried pecan tissues. Each protocol line in the image represents the mean GA concentration (µg/g) for the corresponding tissue and protocol. The value shown above the line is the mean, and the number below indicates the standard deviation (SD).

Fresh samples were ground using a mortar and pestle with liquid nitrogen, heat or freeze-dried samples were ground using an automated grinder followed by a bead beater (**Figure 1**; **Supplementary File**) (Mini-BeadBeater-96, BioSpec Products, Bartlesville, OK, USA). All ground tissue samples were weighed on a precision analytical balance (± 0.00001 g).

### 2.3 GA extraction

Determining an appropriate amount of tissue and a suitable sample processing method is especially needed for ELISA-based quantification of GA in woody plants as their hard tissues with rich metabolites may interfere with the ELISA procedure. However, established GA quantification protocols, particularly for ELISA kits, often assume soft tissue material and require relatively large amounts of material (e.g., 1 g fresh weight), limiting their applicability to small-size and lignified materials like buds, bark, and wood. GA extraction was conducted according to the Cloud Clone protocol (CLOUD-CLONE CORP, Katy, TX, USA) and its four modified versions with changes in the tissue amount, solvent amount, or sample (weight) to solvent (volume) ratio (**Table 1**).

**Table 1:** Original and modified protocols for GA extraction.

Step	Original cloud-clone protocol	Modified protocol A	Modified protocol B	Modified protocol C	Modified protocol D
Tissue weight	~1 g fresh tissue	0.2000 g dried tissue (from ~1 g fresh)	0.0250 g dried tissue (from ~125 mg fresh)	0.0250 g dried tissue (from ~125 mg fresh)	0.0125 g dried tissue (from ~125 mg fresh)
Sample weight: methanol ratio	1:6	1:6	1:40	1:32	1:40
Extraction solvent	6 mL cold 80% methanol	1.2 mL cold 80% methanol	1 mL cold 80% methanol	0.8 mL cold 80% methanol	0.5 mL cold 80% methanol
Vial size	≥8 mL	2 mL	2 mL	2 mL	1.5 mL
Homogenization	Vortex 24h- 4 °C	Same	Same	Same	Same
Centrifugation/filter	4 °C, 3000 g, 10 min or filter	4 °C, 3000 g, 20 min	4 °C, 3000 g, 20 min	4 °C, 3000 g, 20 min	4 °C, 3000 g, 20 min
Solvent separation	Transfer methanol extract into new 8 mL vial	Separating methanol extract into new 2ml vial	Separating methanol extract into new 2ml vial	Separating methanol extract into new 2ml vial	Separating methanol extract into new 1.5ml vial
Second extraction solvent	2 mL cold 80% methanol	0.4 mL cold 80% methanol	0.33 mL cold 80% methanol	0.264 mL cold 80% methanol	0.165 mL cold 80% methanol
Mixing	1 h 4 °C	Same	Same	Same	Same
Centrifuge	4 °C, 2000 g, 10 min	Same	Same	Same	Same
Concentration	Rotary evaporation to dryness 2 ml	–	–	–	–
Phase separation	Add 1.0 mL petroleum ether; remove upper phase	Add 0.2 mL petroleum ether; remove upper phase	Add 0.16 mL petroleum ether; remove upper phase	Add 0.13 mL petroleum ether; remove upper phase	Add 0.16 mL petroleum ether; remove upper phase
Final use	Store overnight at 4 °C or use 50 µL per ELISA well	Same	Same	Same	Same

1 Methanol and petroleum ether volumes were adjusted based on tissue weight. For smaller samples, solvent volumes were scaled accordingly, and final volumes were increased by a factor of 6.6 to ensure adequate volume for pipetting during ELISA preparation.

The tissues used were wood, buds and bark. The specified amounts of the fresh or dried tissue samples were homogenized and extracted with cold 80% methanol (≥99.9% purity, catalog no. AC325740010, Thermo Fisher Scientific, Waltham, MA, USA) at the specified tissue-to-solvent ratios (**Table 1**) and mixed by vortexing for 24 h at 4 °C. The homogenates were then centrifuged at 3000g for 10 min at 4 °C. The methanol supernatant was transferred to a new vial. A second extraction was performed on the pellet with cold 80% methanol, followed by mixing for 1 h at 4 °C and centrifugation for 3000g for 10 min at 4 °C (**Table 1**).

Only for fresh samples, the Cloud Clone sample preparation protocol was followed. The combined supernatants were concentrated by the rotary evaporation process to a final volume of 2 mL. To further purify GA, solvent extraction was conducted with 1 mL of petroleum ether (ACS reagent grade, boiling range 40-60 °C, catalog no. E139-4, Fisher Chemical, Fair Lawn, NJ, USA). After discarding the upper phase petroleum ether, the lower methanol fraction was stored overnight at 4 °C or used directly in the ELISA experiment for GA quantification. Extractions using modified protocols skipped the rotary evaporation step (**Table 1**).

Two drying methods, heat-dry and freeze-dry, were employed when conducting the modified protocols A-C. Investigation using the modified protocol D tested the stability of GA in samples stored at -80 °C for 4 days by comparing to the immediately heat-dried samples without storing in -80 °C. Six replications were conducted for testing the effect of extraction protocols, three replications for testing the effect of drying methods, and twelve replications for determining GA concentrations in pecan tissues using Protocol D while checking on the storage method as well.

### 2.4 ELISA for GA quantification

GA concentrations were determined using a competitive ELISA (Cloud-Clone Corp., CEA759Ge, 96 wellformat), following the manufacturer’s instructions (Cloud-Clone Corp., Katy, TX, USA, n.d.). GA_3_ was used as a standard to create a standard curve. Other reagents and washing buffers during GA test were supplied with the ELISA Kit (Cloud-Clone Corp., Katy, TX, USA). absorbance values were recorded at 450nm, and GA_3_ concentrations were calculated using a standard curve constructed on a semi-log plot, with inverse correlation between absorbance and hormone concentration as typical for inhibition-based assays. Also, Gibberellic Acid A_3_ (Thermo Fisher Scientific Inc., Waltham, MA, USA) was used to spiked recovery analysis.

### 2.5 Spiking: assessment of matrix effects and phenolic interference

To assess the matrix effects and analytical precision of the gibberellic acid (GA) ELISA in woody tissue extracts, spike recovery studies were carried out. For a spike recovery test, two different spike levels were selected within the assay calibration range (123.5-10–000 ng/mL).

For spike recovery test, pecan bark tissue samples collected from July 2025 were used with 0.0125g weight for each sample. Two approaches were used to test the spike recovery. In the first approach, samples were spiked using the GA standard provided in the ELISA kit. In the second approach, sample extracts with relatively high GA concentrations were used to spike sample extracts with lower concentrations. Because 50 µL of each sample was directly loaded into the ELISA wells without further dilution in plate, the GA concentration in each spiked extract tube was adjusted to correspond to the target concentration in the well and its replication.

For the kit GA as spiking, as our first approach, we conducted two tests, the first test included one phloem (bark) sample (with weight 0.0125g) with two spike levels (4444 and 2857 ng/mL) prepared from a 10,000 ng/mL GA stock solution along with pure sample (without spike). In the second test, additional sample (along with the same sample from the first test) with two spike levels along with pure samples (without spike) was performed in a separate ELISA run to confirm consistency of the results.

For the second approach with sample-to-sample spiking, extracts with high GA concentrations (3079 ng/ml) were used as spike source (with spike concentrations of 1539.5, 2052.7, 2771.1 ng/mL) for another sample with lower endogenous GA levels (2738 ng/L).

The concentration of GA added to each sample as spike was calculated based on the added GA_3_ concentration (ng/mL) multiplying by the volume of the stock solution added (mL) dividing by the total final volume of the mixture (mL) (Equation 1) this formula also can be used to calculate the C _sample added_ where the V is the volume of sample multiplying to concentration of sample (neat sample concentration reading) divided into the final volume.

(1) $$C_{added\;spike}\left(ng\;ml^{-1}\right)=\frac{V_{GA\;Stock\;added}\left(ml\right)\times C_{GA\;Stock}\;\left(ng\;ml^{-1}\right)}{V_{final}\;\left(ml\right)}$$

The two spike recovery tests were performed to assess the efficiency of the developed method extraction. Based on the following (Equation 2), the Recovery % was calculated.

(2) $$\text{Recovery(\%)}=\frac{\begin{array}{c} {\text{Concentration}}_{spiked\;sample\;}\left(ng\;ml^{-1}\right)-{\text{Concentration}}_{\text{ unspiked sample}} \end{array}\left(ng\;ml^{-1}\right)}{{\text{Concentrationn}}_{added\;spike}\;\left(ng\;ml^{-1}\right)}\times 100$$

Also, the extracts were physically separated by centrifugation at 4 °C to slow the enzymatic oxidation of phenolics or by filtration (Robinson et al., 2017). After 24 hours and 1 hour of the second extraction to clarify the supernatant before filling the plate wells with the sample.

### 2.6 Data analysis

All data were analyzed using the Steel-Dwass nonparametric test due to violations of normality assumptions with JMP pro 18 (JMP Statistical Discovery LLC., 2025). Also, to investigate whether bark or wood GA concentrations could be predictors for bud GA concentration, an analysis performed simple linear regression analyses using bud GA as the dependent variable and bark/wood GA as the independent variables, and this analysis has been applied to all data that follows protocol D methods. The coefficient of variation (CV%) between replicate wells for every sample in a single plate was used to compute intra-assay precision. Illustrations presented in the **Supplementary Materials** were generated using (BioRender, 2025) under an academic license.

## 3 Results and discussion

### 3.1 Result

#### 3.1.1 Reduced tissue amounts and tissue-to-solvent ratio improved GA detection

Our initial experiment using 1g of fresh tissue per sample and the extraction solvent and Reagent A from the ELISA-kit produced solid residues in the 96-well plate, leading to very low GA-concentration readings (0.12-0.21 µg/g). Using 0.2 g dried tissue (equivalent to approximately 1g of fresh tissue) and the same solvent-to-tissue ratio of 1:6 as specified in the original ELISA kit protocol (Protocol A, **Table 1**) produced similar results (**Figure 1**). We then further reduced the amount of dried tissue to 0.025 g (Protocols B and C, **Table 1**) and 0.0125 g (Protocol D, **Table 1**) and the tissue-to-solvent ratio (Protocols B-D, **Table 1**). Sample processing was performed in 2-mL microcentrifuge tubes instead of 8 mL vials, and the rotary evaporation step was omitted, to reduce analyte loss and handling time. Petroleum ether volumes were also scaled down (0.2, 0.16 and 0.13 mL vs. 1 mL, **Table 1**) to be consistent with the reduced extraction volumes. These modifications resulted in significant increases in GA detection (**Figure 1**).

**Figure 1** shows that the sample amounts and perhaps the tissue-to-solvent ratios-significantly affected the detection of GA, with Protocol D producing the highest and most consistent GA values across all tissues (**Figure 1**).

#### 3.1.2 Freeze-drying and heat-drying produced similar results in GA detection

**Figure 2:**
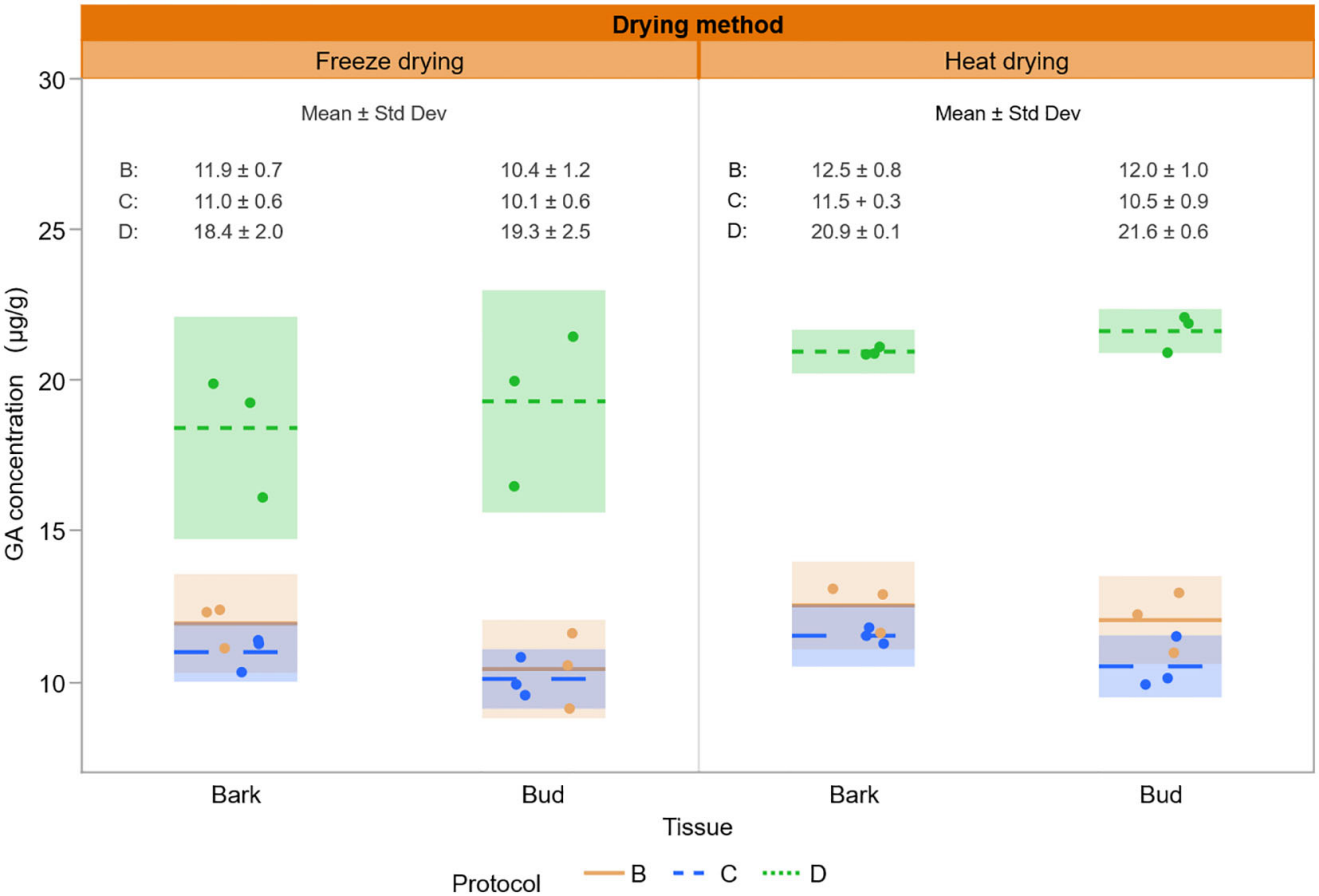
Effect of drying method on GA concentration (µg/g) in different dried pecan tissues for protocols B, C, and D. Each protocol line in the image represents the mean GA concentration (µg/g) for the corresponding tissue and protocol. The value above the line is the mean, and the value below represents the standard deviation (SD).

Comparison of the two drying methods, freeze-drying vs. heat-drying, indicated that GA concentrations did not differ significantly between the freeze-dried samples and the heat-dried samples when either the bud and bark samples were pooled (Steel-Dwass, *p* = 0.5448; **Figure 2**) or separated (bud, *p* = 0.2893; bark, *p* = 0.4799; **Figure 2**). These findings suggest that immediate oven drying did not compromise the GA detection, offering flexibility in sample processing in the laboratory.

#### 3.1.3 Storage at -80 °C did not compromise GA detection

**Figure 3:**
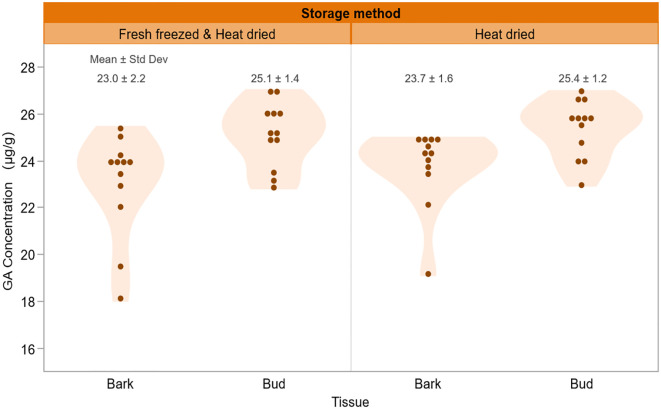
GA concentration (µg/g) in pecan tissues of pecan tree under two storage methods. The contouring area in the violin plot shows the distribution of gibberellic acid (GA) concentrations.

Our experiments also provided an opportunity for testing if storage at -80 °C compromised GA detection when compared with immediately heat-dried samples. Statistical analysis indicated that there was no significant difference in GA concentration between the -80 °C stored and the immediately dried samples (Steel-Dwass, *p* = 0.8703; **Figure 3**). Statistical analysis also indicated that the GA concentration in the bud was higher than the GA concentration in the bark in both the -80 °C stored and the immediately heat-dried samples (Steel-Dwass, *p* < 0.001; **Figure 3**).

**Figure 4:**
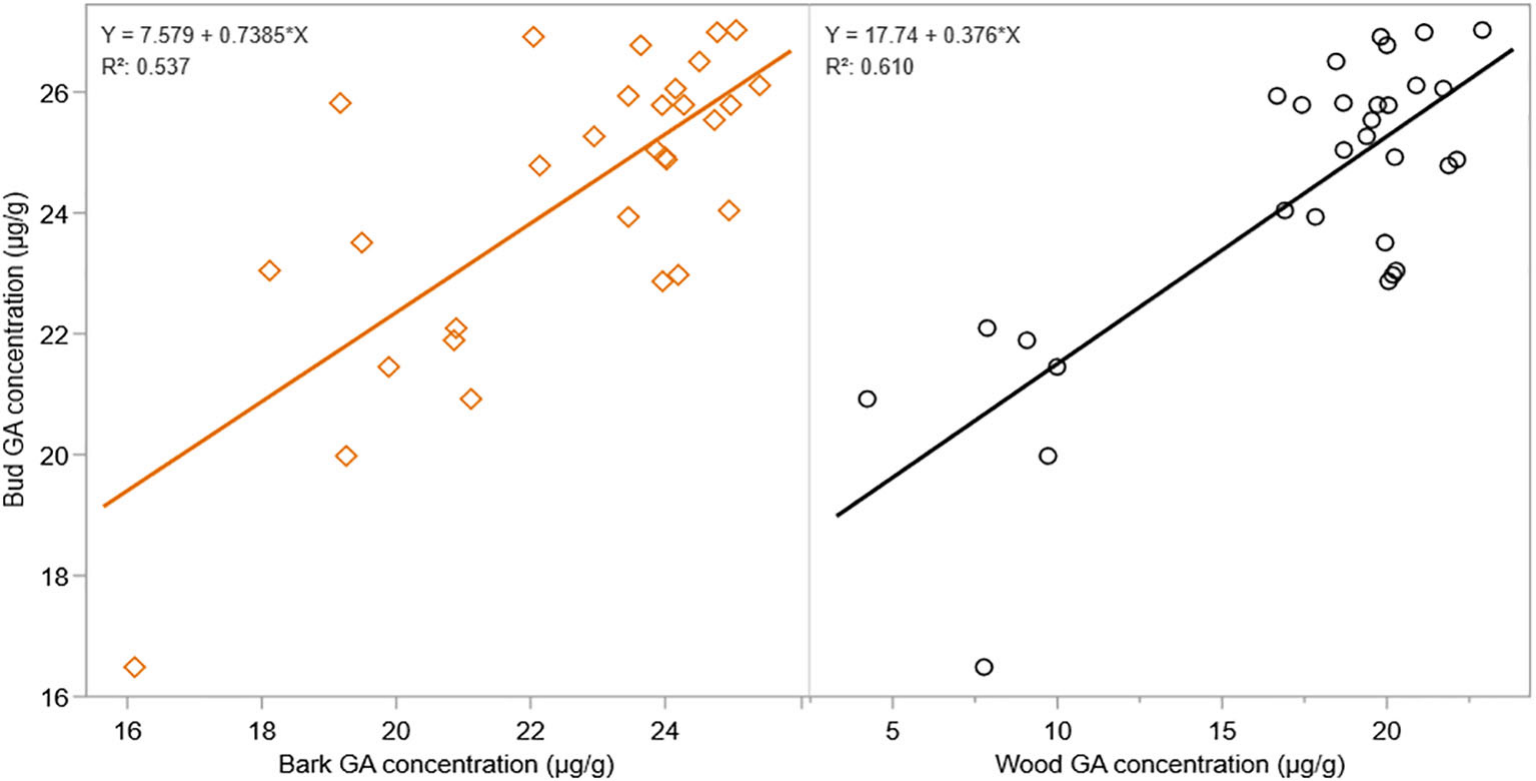
Linear regression analysis showing the association of GA_3_ levels between tissue pairs in protocol D. The left panel (orange) represents [e.g., bud vs. bark], and the right panel (black) represents [e.g., bud vs. wood]. Regression equations and coefficients of determination (R²) are shown in each panel.

According to linear regression analysis GA concentrations in buds and stem tissues showed statistically significant but moderately positive correlations. GA levels in buds were positively correlated with GA concentrations in wood (R² = 0.61, p< 0.0001; **Figure 4**) and bark (R² = 0.54, p< 0.0001; **Figure 4**). These correlations indicate that a major, though incomplete, amount of variation in bud GA levels can be explained by stem GA concentrations. These results showed a considerable correlation between stem GA level and bud GA level rather than an exact quantitative prediction.

Short-term storage of samples at -80 °C for four days before heat-drying did not compromise GA detection, with percent stability of 97.1% in bark, 99.0% in buds, and 104.7% in wood relative to immediately heat-dried samples by using Equation 3 (**Table 2**).

**Table 2:** Stability of GA after short-term storage at -80 °C followed by heat-drying (Protocol D).

Tissue	Storage condition	Mean GA (µg g^-^¹) ± SD	CV (%)	% Stability
*Bark*	-80 °C stored (4 d) + heat-dried	23.03 ± 2.18	9.45	97.1
*Bud*	-80 °C stored (4 d) + heat-dried	25.13 ± 1.40	5.56	99.0
*Wood*	-80 °C stored (4 d) + heat-dried	20.23 ± 1.63	8.08	104.7

2: Percent of stability calculated relative to immediately heat-dried samples for each tissue type. Values represent biological replicates analyzed by ELISA under protocol D. Short-term storage at -80 °C did not substantially reduce GA recovery.

(3) $$\%Relative\text{ stability}=\frac{\begin{array}{c} \left(Mean\;GA\;concentration\;after\;-80{}^{\circ}C\;short\;term\;storage\right) \end{array}}{\text{(Mean GA concentration in immediatly heat-dried sample)}}\times 100$$

#### 3.1.4 Spiked GA was successfully recovered

The acceptable range for the recovery rate is usually 80-120% (Andreasson et al., 2015). The spike recovery test in both approaches (first approach with GA from ELISA kit as spike and second approach with sample as spike to another sample) showed acceptable recoveries (**Table 3**). For the first test with GA as spike (with two levels of spike of 4444 and 2857 ng/mL) added to the sample, showed the 83.69% recovery with higher spiked (4444 ng/mL) and 74.35% with lower spike rate (2857 ng/mL). Similarly in second test with two samples, in the first phloem sample the recovery was 74.56% and 87.07% respectively for the higher GA spike (4444 ng/mL) and lower spike (2857 ng/mL). The second sample had a higher recovery rate with 83.97% and 102.53% recoveries for the high and low spike GA respectively. Similarly, our second approach with sample-to-sample spike showed recovery rates of 81.8%, 117.6%, and 95.16% for the spike concentrations 1539.5, 2052.7, and 2771.1 ng/mL, respectively1720,1985 and 2680 ng/mL was respectively 98%,73%, 111% and 85%. Measured GA_3_ concentrations (ng/mL) are shown for unspiked and spiked samples across different sample IDs and spike levels (2857 and 4444 ng mL^-^¹).

**Table 3:** Spike recovery % from the pecan bark sample using two spike levels and separate ELISA tests using GA (from kit) as a spike (approach 1) and samples to sample spike (as approach 2).

Approach order	Test Group	Sample	Sample (ng/ml)	Concentration of stock of GA/sample used for spike (ng/ml)	GA concentration added (ng/ml) as spike	Spike Recovery %
Approach 1	Test 1	Bark 1	5630.2	10000	4444.0	83.69
			4580.7	10000	2857.0	74.35
	Test 2	Bark 1	4769.4	10000	4444.0	74.56
			4359.0	10000	2857.0	87.07
		Bark 2	5223.1	10000	4444.0	83.97
			4846.7	10000	2857.0	102.53
Approach 2	Test 1	Bark 3	2516.7	3079	20771.1	81.8
			3187.3	3079	2052.7	117.6
			2769.1	3079	1539.5	95.16

* 0.0125 g weight of samples collected in July 2025 was used for all tests for each sample used.

** each recovery % is average of 3–4 reps from each sample.

#### 3.1.5 Method validation

Sample size for intra-assay precision assessment was determined by performing three replicate extractions and ELISA measurements (N = 3) for each tissue×drying method×protocol combination within a single analytical run, which is consistent with standard bioanalytical method-validation practices.

Intra-assay precision was expressed as the coefficient of variation (CV%), calculated as (standard deviation/mean) ×100 for each group. Across protocols B-D, CV values were generally below 15% for bark and bud indicating acceptable repeatability for plant hormone quantification.

In several Protocol-A bud samples, very large CV values were obtained due to mean concentrations approaching zero or being slightly negative after blank correction, which mathematically inflates CV and reflects concentrations near the assay detection limit rather than true analytical instability.

For the storage-method experiment, branch samples were pooled and analyzed together, resulting in 12 replicate samples (N = 12) per tissue and storage treatment within a single assay run. Intra-assay precision was expressed as coefficient of variation (CV%), calculated as (standard deviation/mean) ×100. All pooled storage-method groups show excellent intra-assay precision, with CV values ranging from 4.83-9.45%, well below the commonly accepted 15-20% threshold for plant-hormone ELISA repeatability. Since the same extracts were not used in several analytical runs, inter-assay precision was not assessed.

### 3.2 Discussion

A simple and effective method for quantifying GA_3_ in difficult plant tissues is needed for molecular and physiological investigations. We show here that small tissue amounts (e.g., dried 0.0125 g) and sample heat-drying enabled reliable detection of GA_3_ in pecan bark, wood, and bud tissues by ELISA. Heat-drying can be easily performed, and the dried samples can be safely stored long-term. We also showed that freeze-drying is unnecessary in preserving GA_3_, as heat-drying produced similar results in GA_3_ detection. In addition, consistent with Cao et al.’s work (2022), we demonstrated that freezer storage did not compromise the detection of GA_3_. After all, Protocol D was found to be the most effective protocol for GA_3_ extraction from woody or hard plant tissues.

Also, to prevent phenolic interference, multiple steps, such as the choice of extraction buffer should restrict oxidation and protein phenolic complexes’ interference with the antibody-antigen interaction and color development (Liu et al., 2024). Additionally, phenolic binders (PVPP/PVP) and antioxidants are another effective strategy to limit it (Ranatunge et al., 2017). And centrifugation in 4 °C to slow enzymatic oxidation of phenolics or filtration prior to run (Robinson et al., 2017) can be employed to separate the phenolic compounds in the ELISA. These procedures separately reduce the amount of phenolic matrix in the sample by minimizing both the direct binding of phenolics to antibodies and indirect assay interference to some extent (background color, cross reactivity, and enzyme inhibition).

Our results demonstrated that small quantities of dried pecan tissue could be reliably used for GA_3_ detection via ELISA, eliminating the need for 1 g fresh samples required by the manufacturer’s protocol. Fresh samples pose an additional challenge because some biological processes continue to be active in them after their separation from the plant bodies, possibly leading to degradation or chemical modification of target compounds during handling and analysis (Cha et al., 2009). Evidence from a research suggests that most hormone loss during tissue processing is driven by enzymatic breakdown rather than thermal decomposition at moderate temperatures. The rapid dehydration achieved through controlled heat-drying likely reduced enzymatic activity, thereby to preserve GA content, Shifting to dried tissue analysis offers advantages in storage stability and ease of handling (Dong et al., 2016; Nievola et al., 2017). Woody tissues, with their dense structure, high fiber content, and complex secondary metabolites, are difficult to homogenize and extract when they are fresh (Zhang et al., 2021) So, the drying process breaks down cellular structures, concentrates target analytes by removing water, and reduces the influence of interfering compounds, resulting in more consistent extraction and improved detection limits. Dried tissue can also be ground to a fine powder more effectively, enhancing extraction efficiency and reproducibility. Beyond these analytical benefits, dried samples can be stored at room temperature in sealed containers with desiccants for extended periods (Zhang et al., 2020; Zhang et al., 2021; Wu et al., 2023). Drying facilitates large-scale and comprehensive temporal studies of GA dynamics in pecan development. In short, the drying approach enhances study design flexibility, reduces analytical costs, and supports GA studies involving large numbers of plant samples.

One study showed that UHPLC-MS/MS has enabled comprehensive phytohormone profiling from as little as 0.010-0.020 g fresh weight, and for certain abundant compounds such as GA_24_, less than 0.0001 g is sufficient (Dong et al., 2016). Therefore, the reduced tissue amounts in this study likely reduced matrix interference.

In tea plants, monitoring of GA_3_ profiles across developmental stages revealed that peak GA_3_ levels in stems corresponded with elevated concentrations in buds. The parallel accumulation of GA_3_ between these tissues suggests a coordinated regulatory mechanism in GA production and transportation (Li et al., 2021). Our findings of positive linear correlation in GA concentration between the stem tissues and the bud in pecan align with the findings in tea plants.

## 4 Conclusions

This study optimized the standard ELISA kit protocol for pecan tissues by reducing the dried tissue amount and the tissue-to-solvent ratio. The revised method enabled reliable detection of GA by reducing solid residues, which interfered with spectrophotometer readings in 96-well plates. Heat drying employed in this investigation effectively resolved grinding challenges in lignified or hard tissues. Among all the tissues that have been tested, buds exhibited the highest GA levels. The correlation observed between stem and bud GA concentrations supports the hypothesis that GA concentrations in different parts of a plant are under moderate level of coordinated control of synthesis and transportation.

## Data Availability

The original contributions presented in the study are included in the article/**Supplementary Material**. Further inquiries can be directed to the corresponding authors.
