# Supplementary material for: An effective enzyme-linked immunosorbent assay-based method for quantification of gibberellic acid in pecan (Carya illinoinensis)
Source: Front Plant Sci. 2026 Jun 24;17:1771880. doi: 10.3389/fpls.2026.1771880 (PMC13341426; doi:10.3389/fpls.2026.1771880)
Supplement: Supplementary file 1 [file DataSheet1.pdf]

# Supplementary Protocol D, Optimized ELISA Sample Preparation for Gibberellic Acid (GA) in Pecan Tissues

**Mohadeseh Jahanifard[1], Ming Yang[2]*, Lu Zhang[1]***
[1]Department of Horticulture and Landscape Architecture, Oklahoma State University, Stillwater, OK 74078
[2]Department of Biology, Oklahoma State University, Stillwater, OK 74078
*Corresponding Authors: Lu Zhang, Email: luzhang@okstate.edu; Ming Yang, Email: ming.yang@okstate.edu
Keywords. Bark, Bud, ELISA, GA quantification, Woody plant

## 1. Purpose

This protocol details the optimized method for extracting gibberellic acid (GA) from lignified pecan (*Carya illinoinensis*) tissues for Enzyme-linked immunosorbent assay (ELISA) quantification, using minimum tissue mass and modified solvent ratios to improve detection accuracy.

## 2. Materials

- Tissue: Bud, bark, or wood separated from sampled shoots
- Analytical balance (±0.0001 g)
- Grinder machine
- 2 mL microcentrifuge tubes
- Cold 80% methanol
- Petroleum ether (analytical grade)
- Vortex mixer
- Refrigerated centrifuge capable of 2000–3000 g
- Micropipettes with tips (10–1000 µL)
- Cloud-Clone ELISA kit for GA (CLOUD-CLONE CORP.(CCC), n.d.)
- Refrigerator (4 °C) and −80 °C freezer
- Oven

## 3. Procedure

1. Collect the sample from tree canopy in field need dry ice to transfer it to the laboratory.
2. It can be kept in −80 °C for further processing or just preprocess them immediately.
3. Separating the target tissue (bud, bark, or wood) from the shoot and label them.
4. Immediately heat dry at 60 °C for 4 days in an oven or freeze dry it with for 2 days.
5. Grind dried tissue to a fine powder using grinding machine.
6. Put the grinded material into 2 ml vial in the mini bead beater to get fine powder (figure 1).

## 4. Extraction

### 4.1. First Extraction

1. Weigh exactly 0.0125 g of dried tissue into a 1.5 mL microcentrifuge tube.
2. Add 0.5 mL of cold 80% methanol (1:40 sample to solvent ratio).
3. Vortex the sample for 24 h at 4 °C.
4. Centrifuge at 3000 g for 20 min at 4 °C.
5. Transfer the methanol supernatant to a new 2 mL tube.
   - One important factor in preventing solid particles from mixing with the supernatant after centrifugation is to pipette only from the very top layer (figure 2).

### 4.2. Second Extraction

6. Add 0.165 mL cold 80% methanol to the pellet.
7. Vortex for 1 h at 4 °C.

8. Centrifuge at 2000 g for 10 min at 4 °C.
9. Combine both methanol supernatants.

### 5. Phase Separation

1. Add 0.16 mL petroleum ether to the combined methanol extract.
2. Allow layers to separate; remove and discard the upper petroleum ether phase.
3. Retain the lower methanol phase for ELISA (figure 3).

### 6. Storage and Analysis

- Store extracts at 4 °C overnight if not analyzing immediately.
- Pipette 50 µL per well for ELISA following the manufacturer's assay instructions.

### 7. Note:

This protocol was optimized from the manufacturer's original method (CLOUD-CLONE CORP.(CCC), n.d.) by reducing tissue mass, increasing methanol to sample ratio, and decreasing total solvent volumes to minimize matrix interference and improve GA detection in small, lignified samples.

### 8. References


CLOUD-CLONE CORP.(CCC) (n.d.). Available at: https://www.cloud-clone.com/products/CEA759Ge.html (Accessed July 17, 2025).


### 9. Figures

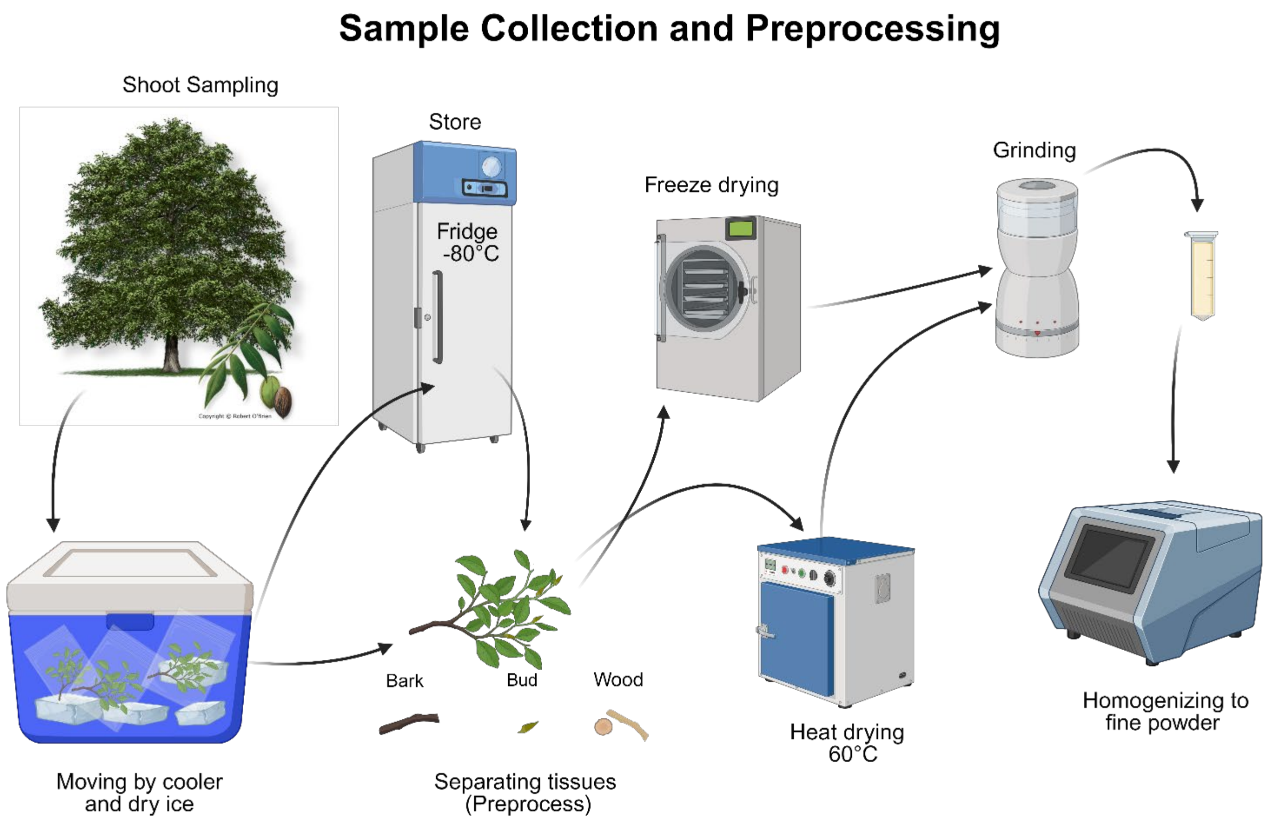


Figure 1. Modified Protocol D Sample collection.

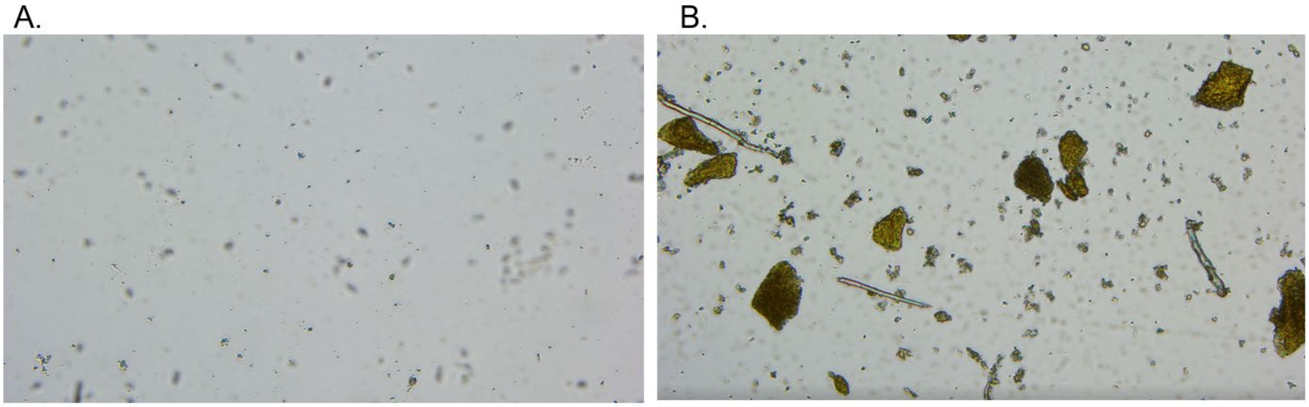


Figure 2. two ways of Supernatant transferring with a pipette of first extraction under Microscope. Note: As shown in the images above, the sample on the left (A) was pipetted carefully from the upper layer, while the sample on the right (B) was taken too close to the powder which is filled with the solids. Therefore, when transferring the incubated methanol into a new vial, this precaution is needed.

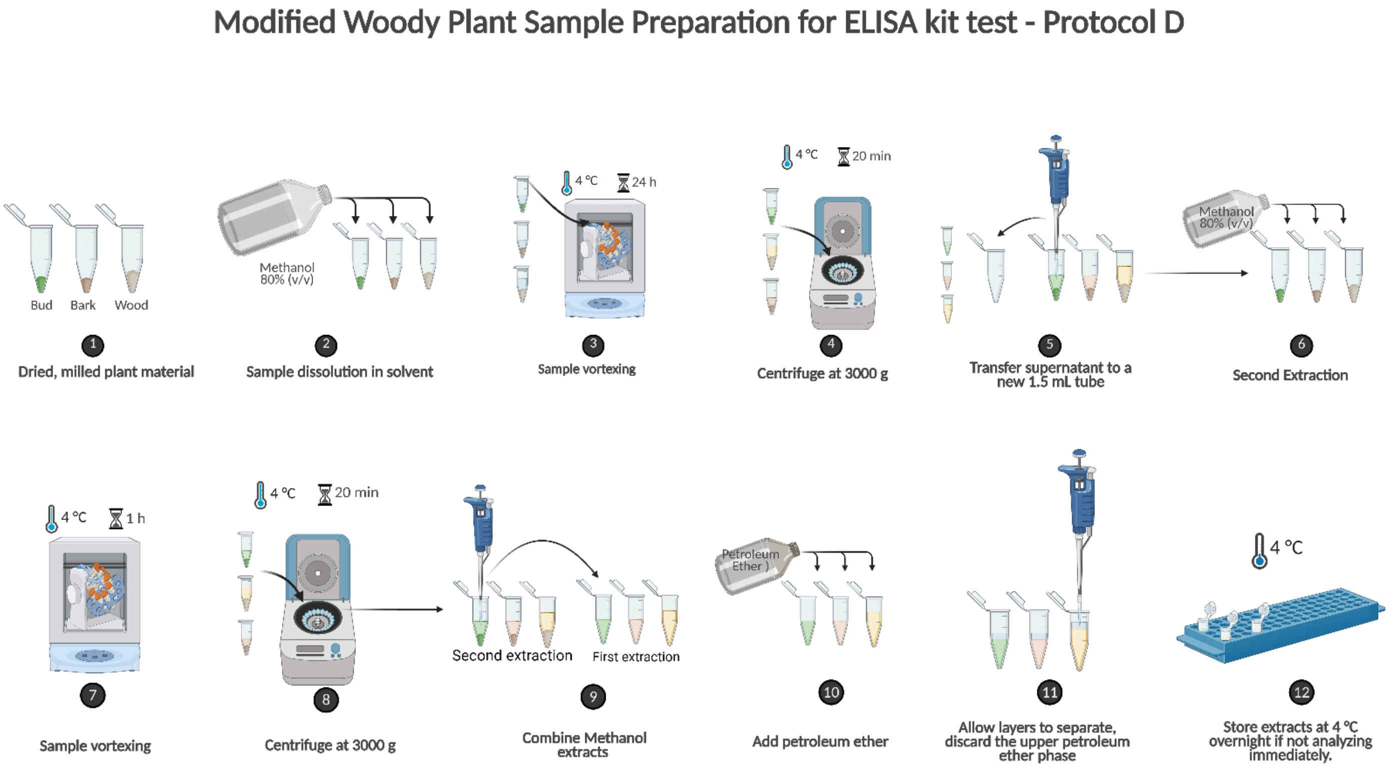


Figure 3. Figure 7 Modified Protocol D Sample preparation.
